# MEK Inhibitors Reverse cAMP-Mediated Anxiety in Zebrafish

**DOI:** 10.1016/j.chembiol.2015.08.010

**Published:** 2015-10-22

**Authors:** Pia R. Lundegaard, Corina Anastasaki, Nicola J. Grant, Rowland R. Sillito, Judith Zich, Zhiqiang Zeng, Karthika Paranthaman, Anders Peter Larsen, J. Douglas Armstrong, David J. Porteous, E. Elizabeth Patton

**Affiliations:** 1 MRC Human Genetics Unit, University of Edinburgh, Edinburgh EH4 2XU, UK; 2 Edinburgh Cancer Research Centre, University of Edinburgh, Edinburgh EH4 2XU, UK; 3 Centre for Genomic and Experimental Medicine, University of Edinburgh, Edinburgh EH4 2XU, UK; 4 Institute of Genetics and Molecular Medicine, University of Edinburgh, Edinburgh EH4 2XU, UK; 5 Department of Biomedical Sciences, Danish National Research Foundation Centre for Cardiac Arrhythmia, University of Copenhagen, 2200 Copenhagen, Denmark; 6 Actual Analytics Ltd, 2.05 Wilkie Building, 22-23 Teviot Row, Edinburgh EH8 9AG, UK; 7 School of Informatics, Institute for Adaptive and Neural Computation, Informatics Forum, University of Edinburgh, Edinburgh EH8 9AB, UK

## Abstract

Altered phosphodiesterase (PDE)-cyclic AMP (cAMP) activity is frequently associated with anxiety disorders, but current therapies act by reducing neuronal excitability rather than targeting PDE-cAMP-mediated signaling pathways. Here, we report the novel repositioning of anti-cancer MEK inhibitors as anxiolytics in a zebrafish model of anxiety-like behaviors. PDE inhibitors or activators of adenylate cyclase cause behaviors consistent with anxiety in larvae and adult zebrafish. Small-molecule screening identifies MEK inhibitors as potent suppressors of cAMP anxiety behaviors in both larvae and adult zebrafish, while causing no anxiolytic behavioral effects on their own. The mechanism underlying cAMP-induced anxiety is via crosstalk to activation of the RAS-MAPK signaling pathway. We propose that targeting crosstalk signaling pathways can be an effective strategy for mental health disorders, and advance the repositioning of MEK inhibitors as behavior stabilizers in the context of increased cAMP.

## Highlights

• PDE4 blockade/AC activation causes novel anxiety-like behaviors in zebrafish
• Small-molecule screen identifies MEK inhibitors as suppressors of cAMP anxiety
• Increased cAMP stimulates crosstalk to RAS-MAPK pathway activity
• New therapeutic potential for MEK inhibitors to treat cAMP-mediated anxiety

## Graphical Abstract

**Figure:**
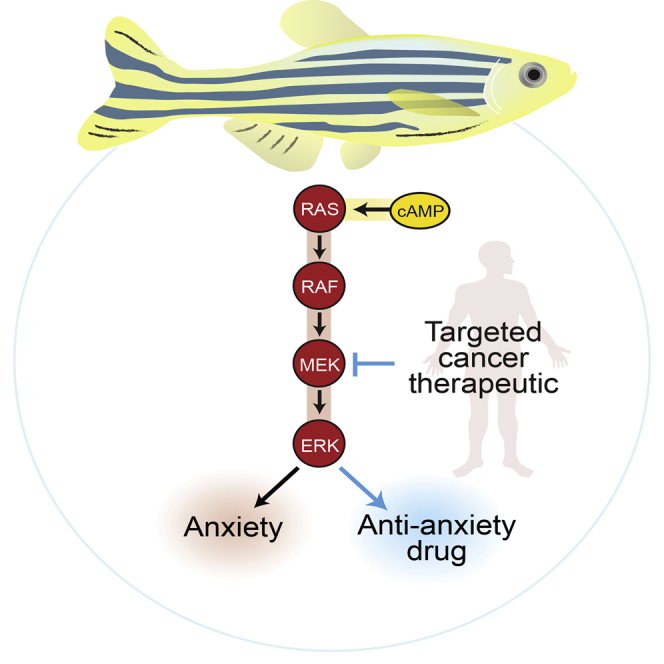

## Teaser

Lundegaard et al. identify therapeutic potential for anti-cancer MEK inhibitors to treat anxiety-like cAMP-mediated behaviors in zebrafish. Targeting cAMP-MAPK crosstalk pathways broadens the range of therapeutic targets for mental health disorders. This work illustrates the importance of whole-animal phenotypic screening in anxiety drug discovery and repurposing.

## Introduction

Mental health conditions afflict one in four adults in their lifetime, with generalized anxiety being the most commonly diagnosed mental health disorder in Western countries (Griebel and Holmes, 2013). There is an urgent need for therapeutic targets and therapies for anxiety, and for the development of new animal models of behavior to be incorporated into anxiolytic drug research (Baldwin, 2011).

The second messengers cyclic AMP (cAMP) and cyclic guanosine monophosphate (cGMP) are critical in the signaling that controls learning, memory, and mood (Maurice et al., 2014, Xu et al., 2011). Intracellular levels of cAMP and cGMP are tightly regulated by tissue-specific phosphodiesterases (PDEs) that catalyze cyclic nucleotide hydrolysis. Genetic and pharmacological evidence indicates that the *PDE4* genes have an important role in controlling cAMP levels in the CNS and behavior (Maurice et al., 2014, Xu et al., 2011). In mammals, PDE4 enzymes comprise four subfamilies (PDE4A–D). Polymorphisms in human *PDE4B* are associated with schizophrenia; we have previously reported complete disruption of the *PDE4B* gene in two independent subjects with psychosis, and that PDE4B and PDE4D interact dynamically with the schizophrenia candidate gene DISC1 to regulate cAMP (Clapcote et al., 2007, Millar et al., 2005, Millar et al., 2007). New PDE4 inhibitors are the focus of intensive drug discovery, not least because recent genome-wide studies indicate that PDE4 may be involved in the pathogenesis of stroke (Nilsson-Ardnor et al., 2005, Staton et al., 2006), bone density (Reneland et al., 2005), and asthma (Hansen et al., 2000, Himes et al., 2009). Underscoring the importance of PDE4 inhibitors in disease, the PDE4 inhibitor rolipram has been reported to have potential as a neuroprotectant, as well as enhance cognition and rescue memory deficits in models of Huntington’s disease, Alzheimer’s disease, diabetes, or following brain injury (DeMarch et al., 2008, Burgin et al., 2010, Cheng et al., 2010, Miao et al., 2015, Titus et al., 2013).

Rolipram is highly specific to PDE4 and is effective in mammals; however, it causes severe emesis in human patients, making it unsuitable as a clinical drug (O'Donnell and Zhang, 2004). In animal studies, pharmacological inhibition of PDE4 can have anti-depressive, sedative, anxiolytic, anti-psychotic, and cognitive enhancing effects, and can increase neurogenesis, but conversely the drug can have anxiogenic effects in some contexts (Burgin et al., 2010, Heaslip and Evans, 1995, Li et al., 2009, Rutten et al., 2008, Silvestre et al., 1999, Siuciak et al., 2007, Zhang et al., 2002). This range of rolipram-induced behaviors likely reflects the importance of specific PDE4 subtypes in regulating distinct behaviors: genetic studies in mice have revealed that anxiety is largely regulated by PDE4A and PDE4B, psychosis by PDE4B, and depression and cognition by PDE4D (Hansen et al., 2014, Li et al., 2011, Siuciak et al., 2007, Siuciak et al., 2008, Zhang et al., 2002, Zhang et al., 2008). DISC1 and PDE4B may also be important in the development of depression caused by chronic stress (Zhang et al., 2015). Notably, the anti-psychotic effects of rolipram and the dependence of these effects on PDE4B are consistent with the association of PDE4B gene disruptions with schizophrenia (Millar et al., 2005, Siuciak et al., 2007, Zhang et al., 2008).

While strong genetic evidence in mice indicates the importance of PDE4A/B in anxiety (Hansen et al., 2014, Zhang et al., 2008), the mechanism through which PDE4-cAMP leads to anxiety remains unknown while being critical for the development of new therapeutic approaches and targets. In zebrafish, PDE4 inhibitors promote anxiety-like behaviors, including decreased habituation to the startle response, increased activity, and thigmotaxis (wall-hugging) in larvae and adult fish (Best et al., 2008, Maximino et al., 2011, Richendrfer et al., 2012, Schnorr et al., 2012, Stewart et al., 2011). Here, we develop a zebrafish model for PDE4-cAMP-mediated anxiety-like behaviors, and use this model to discover chemical suppressors of anxiety in an unbiased, whole-animal phenotypic small-molecule screen. Through screening 80 kinase inhibitors, we discover MEK inhibitors (MEKi) to be highly effective anxiolytics for PDE4-cAMP-mediated anxiety-like behavior in larvae and adult zebrafish. Inhibitors of the mitogen-activated protein kinase (MAPK) signaling pathway have been the focus of intense pharmaceutical interest as targeted cancer therapies. We report the effective use of MEKi to treat anxiety-like behaviors in zebrafish, and demonstrate a new potential for the repurposing of MEKi as anxiolytics.

## Results

### PDE Blockade/Adenylate Cyclase Activation Induces a Novel Anxiety Response in Zebrafish

**Figure 1:**
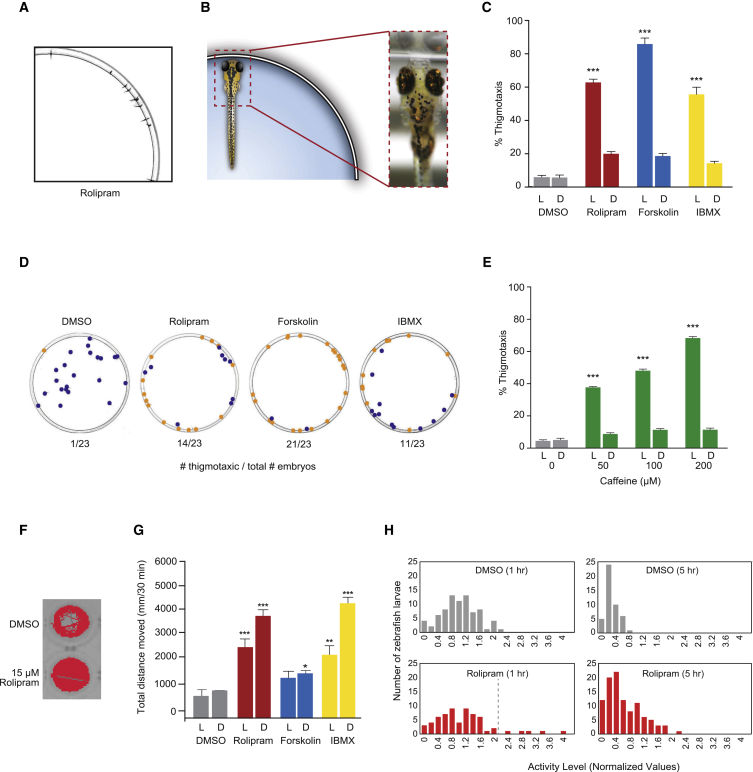
PDE Blockade/AC Activation Stimulates Thigmotaxis and Hyperactivity in Zebrafish Larvae (A and B) Images and schematic representation of thigmotaxis in an open arena following PDE4 blockade/AC activation. (C and D) Quantification (C) and representative image (D) of zebrafish larvae (3 dpf) in 10-cm Petri dish. Larvae exhibiting thigmotaxis are highlighted with orange dots, and non-thigmotaxic larvae with blue dots. Experimental repetitions: n = 7 with 24–35 larvae per treatment condition. Drug treatment concentrations: rolipram 15 μM, forskolin 7.5 μM, IBMX 30 μM. (E) Thigmotaxic response following caffeine treatment. Experimental repetitions: n = 3 (in light and dark) with 35–43 larvae per treatment. (F) Images of individual zebrafish larval traces (1 hr of tracking) in 96-well plate wells. (G) Swimming activity of zebrafish larvae (5 dpf) treated with PDE4 blockers/cAMP activators in light or dark conditions (1 hr treatment, 30 min tracking in the light or dark). Experimental repetitions: n = 4 with 24 larvae per treatment condition. Drug treatment concentrations: rolipram 15 μM, forskolin 7.5 μM, IBMX 30 μM. (H) Histograms showing distribution of total distance moved (normalized values) in response to DMSO (larvae, n = 48) and rolipram (larvae, n = 48), with swimming activity followed over 5 hr. The last 10 min of the first and fifth hour are plotted. Dashed line indicates the approximate separation between the two populations. L, light conditions; D, dark conditions. ^∗^p < 0.05, ^∗∗^p < 0.01, ^∗∗∗^p < 0.001 (one-way ANOVA; Tukey’s multiple comparison test). Error bars denote SEM. See also Movies S1 and S2; Figure S1.

We previously identified disruption of *PDE4B* in two cousins, one diagnosed with schizophrenia and the other with psychosis (Millar et al., 2005). To gain insight into the role of PDE4 in behavior, we modeled the effects of PDE4 inhibition in zebrafish, and developed a small-molecule phenotypic screening platform for novel treatments of PDE4-associated behaviors. Zebrafish are a relevant model system for the study of behavioral disorders because they share homologous molecular and genetic mechanisms with humans, are amenable to genetic and chemical screening, and display defined behaviors, including clear indicators of anxiety and fear (Egan et al., 2009, Blaser et al., 2010, Norton and Bally-Cuif, 2010, Rihel and Schier, 2012, Stewart et al., 2011, Wolman and Granato, 2012). Zebrafish embryos were placed in an open arena (10-cm Petri dish with 30 ml of E3 fish water) and their position was recorded when treated with the PDE inhibitors rolipram or 3-isobutyl-1-methylxanthine (IBMX), or the adenylate cyclase (AC) activator, forskolin (Figures 1A–1D). Rolipram-, IBMX-, or forskolin-treated embryos showed a dramatic and significant thigmotaxic response, whereby the fish swam rapidly to the edge of the dish (Figures 1A–1D; Movies S1 and S2; Figure S1). Unlike other described zebrafish thigmotaxis assays (Richendrfer et al., 2012, Schnorr et al., 2012), there was no experimental trigger in the open arena. Close examination of the fish at the edge of the dish revealed a shadow at the rim of the dish (Figure 1A). We found that the thigmotaxic behavior was dependent on the ability of the fish to detect differences in light conditions (Figure 1C). Caffeine, a well-established anxiogenic drug in mammals and zebrafish and a non-selective PDE inhibitor, was used as a positive control (Figure 1E), providing corroborating evidence that the open-field thigmotaxic response is an anxiety-like behavior in zebrafish larvae.

### PDE Blockade/AC Activation Induces Hyperactivity

We hypothesized that PDE blockade/AC activation could stimulate additional anxiety-like behaviors in zebrafish, such as hyperactivity, as measured by increased swimming activity. Zebrafish larvae were arrayed individually into wells of a 96-well plate, treated with compound for 1 hr, and swimming activity was tracked and recorded for a further hour (Figure 1F). Zebrafish treated with PDE blockade/AC activation agents showed significantly enhanced activity in both the light and the dark (Figures 1F–1H). Notably, rolipram-induced hyperactivity was sustained over 5 hr (Figure 1H). Comparison of the data distribution histograms in Figure 1H for DMSO-treated zebrafish and rolipram-treated fish revealed a complex response to rolipram. Not only did rolipram significantly increase the mean activity level over 1 hr in the dark compared with DMSO-treated fish, it also divided fish into two distinct populations (dashed line in Figure 1H; see also Experimental Procedures).

### Small-Molecule Screening for Chemical Suppressors of Anxiety-like Behaviors

**Figure 2:**
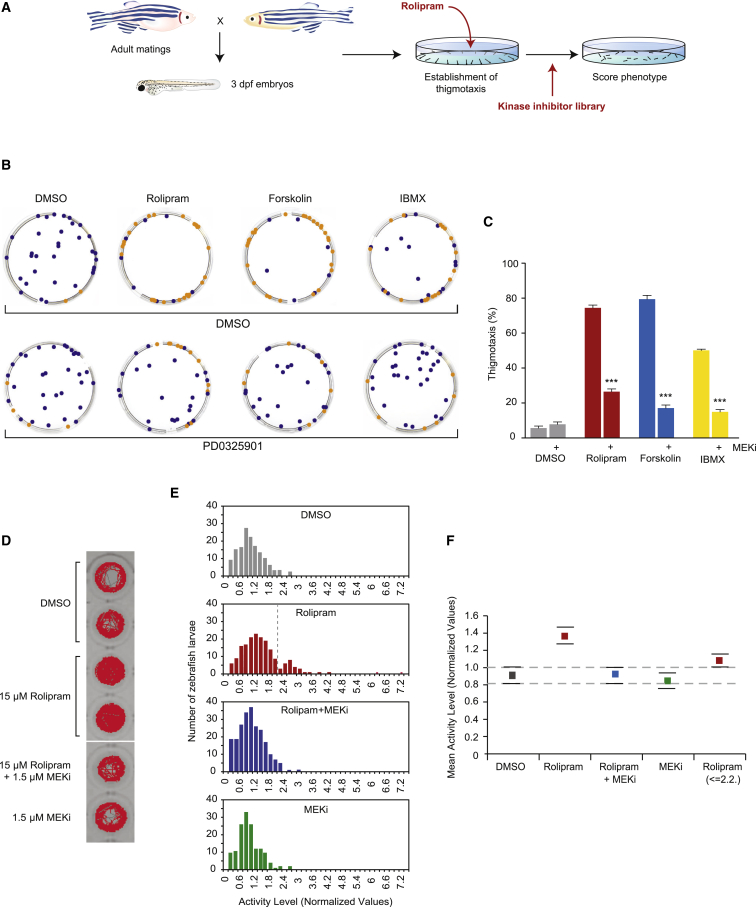
MEKi Reverse Behaviors Caused by PDE4 Blockade/AC Activation (A) Schematic overview of small-molecule screen for suppressors of thigmotaxic behavior. (B and C) Images (B) and quantification (C) of behavior of zebrafish larvae in an open arena. Orange dots: thigmotaxic behavior; blue dots: non-thigmotaxic behavior. Experimental repetitions: n = 6/7 with 25–35 larvae per treatment condition. (D) Images of individual zebrafish larval traces in 96-well plate. Zebrafish were treated for 1 hr with rolipram followed by 1 hr treatment with or without MEKi, and tracked for 1 hr. (E) Histograms showing distribution of total distance moved (normalized values) in response to treatment conditions: DMSO (n = 143 larvae), rolipram (n = 185 larvae), rolipram with MEKi PD0325901 (n = 227 larvae), and MEKi PD0325901 (n = 149 larvae). (F) Total distance moved (normalized values from Figure 2E). Means and bootstrapped 95% CIs are shown. Dotted lines indicate the upper and lower CI limits for the DMSO-treated fish to facilitate comparison with this group. Due to the non-normal distribution and unequal variance of data across groups, means were compared by assessing the bootstrapped 95% CI of the differences between groups. Drug treatment concentrations: rolipram 15 μM, forskolin 7.5 μM, IBMX 30 μM, MEKi 1.5 μM. ^∗^p < 0.05, ^∗∗^p < 0.01, ^∗∗∗^p < 0.001 (one-way ANOVA; Tukey’s multiple comparison test). Error bars denote SEM.

Having established a robust thigmotaxic response to PDE4 blockade/cAMP activation, we wanted to identify small molecules that could restore normal behavior. Eighty kinase inhibitors taken from the Screen-Well Kinase Inhibitor Library (EnzoLifeSciences) were screened to identify compounds that could reverse the rolipram-induced thigmotaxic response. First, zebrafish larvae were treated with 15 μM rolipram to evoke the thigmotaxic response. Next, 15 μM of each of the kinase inhibitors was added to the water, the plate was swirled to ensure even distribution of the inhibitor in the water, and the position of the fish was recorded 1 hr later. Control-treated embryos rapidly reestablished themselves at the edge of the dish (Figure 2B). The most potent thigmotaxis inhibitors were the MEKi PD98059, and the growth factor receptor inhibitors Tyrphostin 25 and Tyrphostin AG-370.

Given the potent effect of the MEKi PD98059 in our screen, we tested the effect of PD0325901, a chemically divergent, clinically active and highly specific MEKi (Samatar and Poulikakos, 2014) on PDE4 blockade/cAMP activation (Figures 2B and 2C). Addition of 1 μM PD0325901 significantly rescued the thigmotaxic effects of PDE4 blockade/cAMP activation within 30–40 min. These results were corroborated by the results from the activity assay (Figures 2D–2F). As in Figure 1H, rolipram treatment divided fish into two distinct populations made apparent by two peaks in the distribution histogram (Figure 2E). MEKi treatment was anxiolytic for rolipram-induced hyperactivity (Figures 2D–2F), supporting a role for MEKi in treating anxiety.

### cAMP-RAS-MAPK Pathway Crosstalk in Anxiety

**Figure 3:**
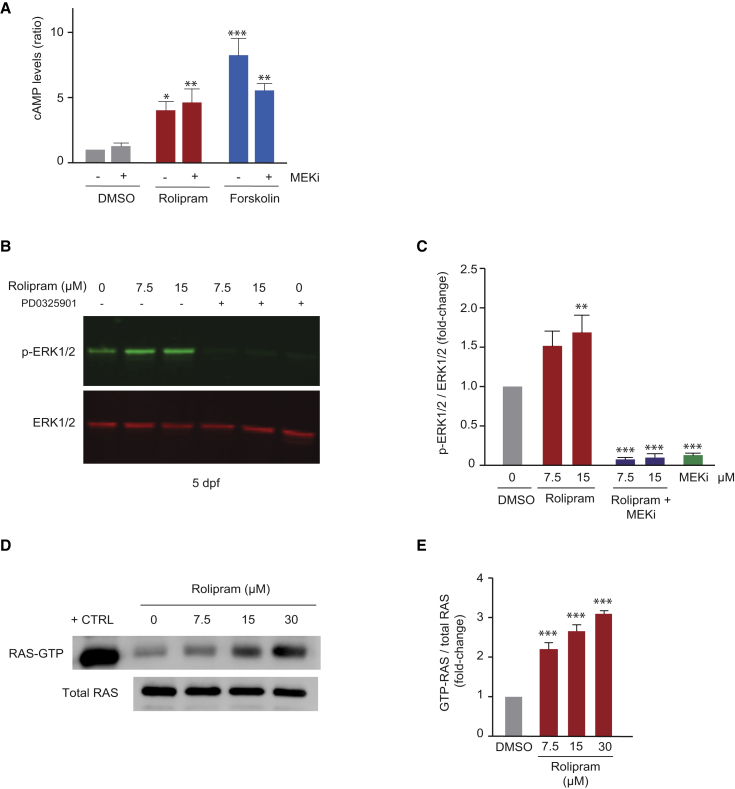
Crosstalk between cAMP and ERK Signaling Pathways (A) Total cAMP concentration in zebrafish larvae (5 dpf) following treatment with rolipram (15 μM) or forskolin (7.5 μM) (1hr), followed by treatment with or without MEKi PD0325901 (1.5 μM) (1 hr). Experimental repetitions: n = 3. ^∗^p < 0.05, ^∗∗^p < 0.01, ^∗∗∗^p < 0.001 (one-way ANOVA, Tukey’s multiple comparison test). (B and C) Western blotting (B) and quantification (C) of zebrafish extracts following treatment with rolipram (1 hr) and/or 1.5 μM MEKi PD0325901 (1 hr), and probed with antibodies to ERK1/2 and phospho-ERK1/2. Experimental repetitions: n = 4. ^∗∗^p < 0.001 (one-way ANOVA; Tukey’s multiple comparison test). (D and E) Western blotting (D) and quantification (E) of levels of active RAS (RAS-GTP) following immunoprecipitation with Raf-1-bound agarose beads. Probed with antibodies to RAS-GTP and RAS. Experimental repetitions: n = 3 (n = 2 for 30 μM). ^∗∗∗^p < 0.05 (one-way ANOVA; Tukey’s multiple comparison test). Error bars denote SEM.

Because of the sensitivity of PDE4 blockade/cAMP activation anxiety-like phenotypes to MEKi, we hypothesized that the underlying molecular mechanism was via activation of the MAPK signaling pathway. First, we confirmed that rolipram and forskolin treatment led to increased cAMP levels in zebrafish larvae, and that these remained significantly increased in embryos co-treated with MEKi (Figure 3A). Next, MAPK pathway activity was investigated by collecting zebrafish embryos treated with rolipram for an hour, then treated with or without MEKi for another hour, and western blotting for phospho-ERK and ERK proteins. Importantly, as shown in Figure 3B, a significant increase in phospho-ERK signal was clearly detected upon rolipram treatment in 5-dpf (days post fertilization) embryos (Figures 3B and 3C), revealing that rolipram treatment leads to activation of the MAPK signaling pathway in zebrafish larvae. Co-treatment with MEKi abolished MAPK pathway activation (Figures 3B and 3C), correlating with the restored behaviors observed in the PDE4 blockade-MEKi combined treatment regimes. To confirm the specificity and establish the molecular mechanism through which rolipram regulates ERK signaling, we assayed RAS activity after rolipram treatment of 5-dpf embryos. Similar to ERK1/2 phosphorylation, we found a significant increase in active RAS (RAS-GTP) following dose-dependent rolipram treatment (Figures 3D and 3E). Collectively, our results demonstrate that increasing doses of rolipram promote cAMP-RAS-MAPK pathway activation.

### MEKi Submaximal On-Target Activity to Treat Anxiety

**Figure 4:**
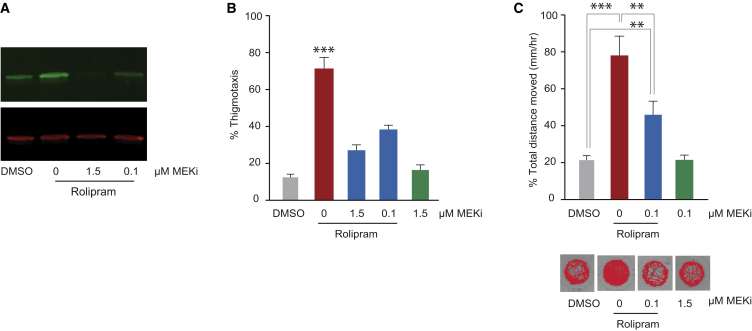
MEKi Treatments at Submaximal On-Target Concentrations Relieve Anxiety (A) Western blotting of zebrafish extracts following treatment with rolipram (1 hr) and/or MEKi PD0325901 at 1.5 μM or 0.1 μM (1 hr), and probed with antibodies to ERK1/2 and phospho-ERK1/2. Experimental repetitions: n = 3. (B) Thigmotaxis assay (3 dpf) with 15 μM rolipram and/or 1.5 μM or 0.1 μM MEKi treatment. Experimental repetitions: n = 3. (C) Swimming activity of zebrafish larvae (5 dpf) following treatment with rolipram (1 hr) with or without MEKi (1 hr). Images of individual zebrafish larval traces from representative wells in a 96-well plate (1 hr). Experimental repetitions: n = 2 with 24 larvae per treatment group. ^∗∗^p < 0.01; ^∗∗∗^p < 0.001 (one-way ANOVA; Tukey’s multiple comparison test). Error bars denote SEM.

MEKi are potent targeted therapies designed to effectively shut off the MAPK signaling in cancers with activating mutations in RAS and BRAF (Samatar and Poulikakos, 2014). We wanted to test whether full MAPK pathway inhibition was essential to treat anxiety-like behaviors, or whether anxiolytic effects could be induced by partial on-target pathway inhibition. Zebrafish embryos were treated with rolipram and 0.1 μM MEKi, a 15-fold dose reduction compared with experiments presented in Figures 2 and 3. Western blotting of zebrafish lysates indicated that 0.1 μM MEKi partially inhibits phospho-ERK1/2 (67% phospho-ERK compared with levels in the DMSO treatment; Figure 4A). Anxiolytic effects of 0.1 μM MEKi on rolipram-treated embryos were evident in both the thigmotaxis assay (3 dpf; Figure 4B) and the larval activity assay (5 dpf; Figure 4C). Thus, partial inhibition of the MAPK pathway is effective in ameliorating anxiety-like behavior, albeit less effectively than at higher doses, and reveals potential for MEKi treatments to be customized for variable pathway inhibition depending on the disease context.

### MEKi Treatment for Anxiety in Adult Zebrafish

**Figure 5:**
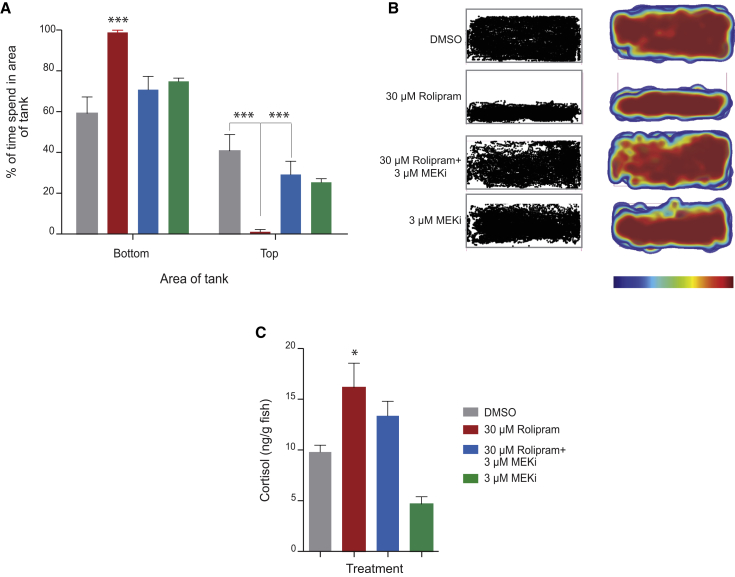
MEKi Ameliorate Anxiety Behaviors in Adult Zebrafish (A) Group behavior assay of adult zebrafish, following 20-min incubation in 1 l of system water with indicated treatments. Behavior was recorded from the side and the distribution in the tank analyzed. Experimental repetitions: n = 6 with 25 adult fish per treatment condition. ^∗∗∗^p < 0.001 (two-way ANOVA; Bonferroni post-test). (B) Bird-nest representations of the tracks of groups of five adult zebrafish taken from the side of a 1-l tank, and activity heatmap corresponding to relative amount of time spent by the fish in areas of the tank during a 5-min period. Maps are 2D Kernel Density Estimates derived from animal coordinates, rendered with a custom color map to enhance visibility: clear (none/minimal), blue (low), through to red (high). Experimental repetitions: n = 6. (C) Cortisol levels of treated adult zebrafish (n = 5 individual fish). ^∗^p < 0.01 (one-way ANOVA; Tukey’s multiple comparison test). Error bars denote SEM. See also Figure S2.

To increase the translational impact of our finding, we tested whether MEKi treatment could alleviate anxiety-like behaviors due to PDE4 blockade in adult zebrafish, rather than during the stages of active neuronal development. We tracked groups of five adult sibling zebrafish from the side view for their placement preference in a tank with 1.0 l of water (group behavior assay; n = 6 experimental replicates) (Figures 5A and 5B). Rolipram-treated fish showed a distinct preference for the bottom of their container, consistent with increased anxiety (Figures 5A and 5B). Critically, MEKi treatment restored the normal tank distribution of rolipram-treated adult zebrafish compared with PDE4 blockade alone (Figures 5A and 5B). Rolipram-treated zebrafish had increased levels of cortisol, an indicator of increased levels of stress, which was significantly reduced upon addition of MEKi (Figure 5C).

Notably, unlike currently available anxiolytics, yet consistent with larval studies in Figures 2, 3, and 4, we found no evidence for MEKi to exert anxiolytic effects alone in adult zebrafish. This was further supported in the novel tank assay (Egan et al., 2009), whereby single adult zebrafish were tracked for 5 min when placed in a novel tank (3 l) and compared with the anxiolytic buspirone (a serotonin receptor 5-HT_1A_ partial agonist) and rolipram as an anxiogenic. Fish treated with the anti-anxiety drug buspirone displayed anxiolytic effects, such as reduced time to explore the upper regions of the tank, while MEKi treatment alone had no effect on zebrafish behavior (Figure S2). Thus, we find that MEKi are potent anxiolytics in adult zebrafish with cAMP-mediated anxiety, but show no anxiolytic effects when used as single agents.

## Discussion

**Figure 6:**
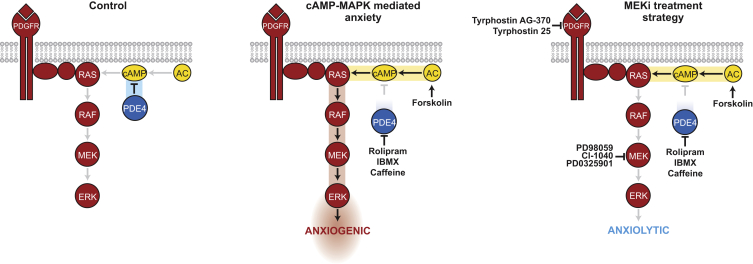
A Novel Therapeutic Strategy: MEK Inhibitors to Treat cAMP-Mediated Anxiety Schematic for the repositioning of MEKi as novel therapeutics for PDE4 blockade/AC activation anxiety. In control fish, PDE4 is a negative regulator of cAMP activity. PDE4 blockade/AC activation leads to increased cAMP activity and activation of the MAPK signaling pathway, and is anxiogenic. Treatment with MEKi reduces MAPK activity and is anxiolytic.

Both genetic and environmental factors contribute to anxiety and depression disorders. Human genetics and pharmacology indicate that PDE4 has an important function in behavior. However, treatment for anxiety is centered on sustaining signaling in postsynaptic neurons, and these treatments vary in their effectiveness and are often associated with unwanted side effects (Baldwin, 2011, Griebel and Holmes, 2013, Maurice et al., 2014). Here, we have developed an alternative strategy to identify context-specific anxiolytics by first developing a zebrafish model for PDE4 blockade/cAMP anxiety-like behavior and then undertaking an in vivo phenotypic screen for suppressors of zebrafish anxiety behaviors. We discover that treatment with MEKi, originally developed as anti-cancer agents, are effective anxiolytics in the context of PDE4 blockade/cAMP activity (Figure 6). Rolipram-induced anxiety-like behaviors lead to an increase in GTP-bound (active) RAS and phospho-ERK levels in zebrafish embryos (Figures 3C and 3D). MEKi treatment leads to a reduction in phospho-ERK levels and anxiety-like behaviors (Figures 2, 3, 4, and 5). Importantly, unlike clinically active anxiolytics such as buspirone, our studies here indicate that the MEKi have no anxiolytic effects alone (Figures 2, 3, 4, 5, and S2), suggesting that they may be an improvement over current therapeutic approaches for anxiety.

In mice, regulation of anxiety is largely mediated by PDE4A and PDE4B (Hansen et al., 2014, Zhang et al., 2008). Here, we hypothesize that the anxiety-like behaviors of rolipram in zebrafish are mediated through inhibition of Pde4A/B because: (1) mouse and zebrafish PDE4A/B genes are highly conserved (Pde4a: 73% identity; Pde4ba: 75% identity; Pde4bb: 72% identity); (2) the anxiety-like behaviors of rolipram in zebrafish are consistent with the anxiety phenotypes of *pde4a* and *pde4b* mutant mice (Hansen et al., 2014, Zhang et al., 2008); and (3) rolipram in zebrafish leads to an increase in cortisol level, which is also consistent with increased plasma corticosterone levels in *pde4a* and *pde4b* mouse genetic mutants (Hansen et al., 2014, Zhang et al., 2008). Interestingly, unlike PDE4B, PDE4A lacks an ERK phosphorylation site (Baillie et al., 2000, Zhang, 2009), suggesting that PDE4B may have the potential to be directly regulated by MAPK signaling. Finally, in contrast to the effects of rolipram treatment, our genetic studies indicate that adult *pde4d*^*−/−*^ zebrafish mutants have anxiolytic behaviors, including an increase in shoal size and increased tank exploration, compared with *pde4d*^*+/+*^ wild-type siblings (Figures S3B and S3C). In addition, *pde4d*^*−/−*^ mutants respond to the anxiogenic effects of rolipram (Figure S3E), indicating that the targets of rolipram in anxiety are via other PDE4 subfamilies and not PDE4D.

Rolipram also has sedative effects in mice that may be linked to inhibition of PDE4D (e.g. Hu et al., 2011). We did not observe sedative effects in zebrafish, possibly because the primary rolipram PDE4 targets in zebrafish are more likely PDE4A or PDE4B; however, we do find that *pde4d* mutant fish swim less overall distance compared with sibling controls (Figure S3D). Interestingly, some larvae appear to be hyper-responsive to rolipram in the activity assay (Figures 1H and 2E). At this stage, we do not know whether this reflects subpopulations of zebrafish with differences in *PDE4* expression and regulation of anxiety, or whether there are individual differences in drug uptake and metabolism. Further spatial and temporal expression studies on *PDE4* subfamilies, coupled with genetic mutants, will be important in dissecting PDE4 functions in anxiety. For example, *PDE4B* expression in mice is highest in regions of the brain associated with anxiety (Cherry and Davis, 1999, Engels et al., 1995, Perez-Torres et al., 2000, Zhang et al., 2008).

An important aspect of drug development is the repositioning (repurposing) of available drugs used to treat one disease for the treatment of a different disease. The MAPK pathway has been shown to interact with PDE4D (Hoffmann et al., 1999, Sheppard et al., 2014, Zhang et al., 2004), but to date has not been associated with other PDE4 subfamilies and anxiety, providing new opportunities for treating anxiety. Highly selective MEKi are currently in clinical trial for ovarian and thyroid cancer, and for use alone or in combination with BRAF^V600E^ inhibitors in melanoma (Samatar and Poulikakos, 2014). We, and others, have previously proposed that MAPK pathway inhibitors may hold promise for the management of syndromes with germ-line activating mutations in the RAS-MAPK pathway (the RASopathies) (Anastasaki et al., 2009, Anastasaki et al., 2012, Pagani et al., 2009, Rauen, 2013, Runtuwene et al., 2011, Tidyman and Rauen, 2009, Viosca et al., 2009, Wolman et al., 2014, Wu et al., 2011). Notably, one of the clinical features associated with the RASopathies is increased anxiety (Axelrad et al., 2011), consistent with our findings that increased MAPK signaling is anxiogenic. Of note, rolipram can reverse memory deficits induced by infusion of the MEKi U0126, suggesting PDE4-MEK crosstalk may also be active in memory formation (Zhang et al., 2004). Based on animal models, the potential for unwanted effects of MEKi on memory may require clinical investigation, although such symptoms have not been reported in cancer clinical trials (trametinib prescribing information, US Food and Drug Administration).

It will be important to compare the effective concentration of MEKi in the context of cancer compared with behavior disorders. In our zebrafish RASopathy models, continuous treatment with low doses of MEKi restored normal development without additional unwanted developmental effects of MAPK pathway inhibition (Anastasaki et al., 2012). For cancer treatment, higher concentrations may be needed to shut off the MAPK pathway, while lower doses may be effective in restoring normal MAPK signaling in the treatment of anxiety. This may have important implications for minimizing the unwanted effects of MAPK pathway inhibition. Here, we find that partial inhibition of MAPK signaling is sufficient to reduce anxiety-like behaviors in zebrafish, indicating that submaximal on-target activity may be efficacious in treating anxiety-like behaviors (Figure 4). However, we observed that low-dose MEKi treatment was not fully efficacious in the activity assay (Figure 5C), suggesting that dysregulation of the MAPK pathway by ectopic activation, in addition to the total levels of MAPK signaling, contribute to anxiety phenotypes.

In conclusion, we present the conceptual advance that targeting the MAPK crosstalk signaling pathway is a novel therapeutic approach to treat PDE4-cAMP-mediated anxiety. We propose that PDE4-MEK crosstalk regulates anxiety-like behaviors, and that highly specific MEKi, originally designed as anti-cancer therapies, may have repositioning potential as anxiolytics (even at submaximal on-target doses) in the context of increased cAMP. Our work underscores the power in drug discovery of unbiased phenotypic small-molecule screening in zebrafish and drug repurposing for mental health disorders.

## Significance

**Anxiety disorders are among the most common mental health disorders, afflicting 10%–20% of the Western adult population during their lifetime. Currently available drug treatments act by dampening neuronal excitability, are limited in their efficacy, and can have unwanted side effects. Here, we propose that the clinically effective MEKi, originally designed to treat cancer, may be repurposed as anti-anxiety drugs for patients with high-cAMP-induced anxiety. First, we developed a model of anxiety-like behaviors by treating zebrafish with inhibitors of PDE4 or activators of cAMP. This model is relevant because cAMP levels play a critical role in behavior, and we have previously shown that disruptions in *PDE4B* are associated with mental health disorders. Next, we performed a small-molecule screen for compounds that can restore normal behavior in our zebrafish anxiety-like model. Phenotypic drug screening is important because it enables us to identity drug leads in an unbiased fashion and reveals new targets in a live animal. We discover that MEKi are highly effective anxiolytics in zebrafish, and that the mechanism of PDE4 blockade/cAMP activation induced anxiety is via stimulation of the RAS-MAPK signaling pathway. cAMP-MAPK signaling crosstalk in anxiety has not been previously reported, and is significant because it reveals that targeting the crosstalk MAPK signaling pathway offers a potent alternative therapeutic strategy for anxiety. Notably, our zebrafish studies indicate that unlike in cancer treatment, MEKi may be effective at low treatment doses that promote only partial, on-target MAPK inhibition, thereby potentially minimizing drug-induced side effects. In conclusion, the conceptual advance we present here is that targeting the crosstalk MEK signaling pathway is a new anxiolytic target, and that anti-cancer MEKi have the potential to be repurposed as anxiolytics for cAMP-mediated anxiety.**

## Experimental Procedures

Zebrafish work was conducted according to the standards and ethical guidelines established by the University of Edinburgh and the Home Office of the United Kingdom (Animal Scientific Procedures Act, 1986), and the University of Copenhagen and the Danish Animal Experiments Inspectorate.

### Drug Treatments, Thigmotaxis Assay, and Small-Molecule Screen

For zebrafish chemical treatments, forskolin, rolipram, and IBMX (all from Tocris Bioscience), caffeine (Sigma-Aldrich), and PD0325901 (University of Dundee, UK) were prepared in DMSO. We noted some batch-to-batch variability for rolipram. For the kinase library screen (EnzoLifeSciences Screen-Well Kinase Inhibitor Library), three dpf wild-type embryos were incubated in 15 μM rolipram in 10-cm Petri plates to establish thigmotaxis, then the kinase inhibitor was added (final concentration 15 μM), the plate swirled, and the dish observed 1 hr later.

### Live Larval 96-Well Tracking

Individual 5-dpf embryos were distributed into a well of a 96-well plate, in 100 μl of E3 embryo medium, and allowed to habituate for 1 hr prior to drug treatment. Following 1 hr of drug treatment, the behavior of the larvae was tracked for another hour in the DanioVison Observation Chamber and the behavior was analyzed using EthoVision XT 8.5 software (Noldus Information Technology). For the swimming activity data shown in Figure 1H, data were pooled across several plates to better examine data distributions. In these experiments data were normalized to the grand average of each individual plate to minimize plate-to-plate variation.

### Western Blots

Following drug treatment, larvae were snap-frozen in liquid nitrogen and protein was extracted using lysis buffer: 20 mM Tris-HCl, 150 mM NaCl, 1 mM EDTA, 1 mM EGTA, 1% Triton X-100, and 30 mM NaF, with PhosStop and CompleteMini inhibitors (Roche). Larvae were disrupted using a hand-held pestle and allowed to incubate on ice for 15 min, prior to spinning at 14,000 rpm for 15 min at 4°C. Protein samples were run on gradient gels (4%–20% TGX-gels; Bio-Rad), and transferred to an Immobilon-FL PVDF membrane (Millipore). Blocking and antibody incubation was done in Odyssey Blocking Buffer diluted 1:1 in PBS. Antibodies were anti-phospho-p42/44 (1:2,000; Cell Signaling Technology) and anti-p42/44 (1:1,000; Cell Signaling Technology). Secondary antibodies were IRDye labeled donkey anti-mouse antibody (1:15,000) and goat anti-rabbit antibody (1:15,000).

### Adult Group Behavior Analysis

Groups of five wild-type fish were incubated for 20 min in 1.0 l of system water plus DMSO or compound, and recorded and tracked (5 min) from the side. Due to batch variability, rolipram was used at concentrations of 20–40 μM. Experiments in Figure 5 were repeated six times (five times as blinded study). Swimming fish were recorded with a Panasonic Lumix DMC-TZ8 camera and each recording tracked using ActualTrack (Actual Analytics).

### Cortisol Determination

Fish from Figures 5A and 5B were immersed in ice-water, the brain tissue removed, and cortisol extracted using diethylether as described previously (Barcellos et al., 2007), with the samples left overnight for ether evaporation as described by Cachat et al. (2010). Following evaporation, cortisol was reconstituted in PBS, and cortisol levels were determined using an ELISA kit (Salimetrics Europe).

### Cyclic AMP Extraction

Zebrafish larvae (5 dpf; n = 15 per treatment per experiment) were snap-frozen in liquid nitrogen. cAMP was extracted by homogenizing samples using a hand-held homogenizer with pestle in 200 μl of 0.1 M HCl. Samples were sonicated for 15 s and spun at 13,000 rpm (15 min at 4°C). Supernatant was transferred to a fresh Eppendorf tube and cAMP concentration was determined (Direct cAMP ELISA kit; Enzo Life Sciences).

### RAS Activity Assays

RAS activity (RAS-GTP) was measured by Raf-1 RBD immunoprecipitation using the RAS Activation Assay Kit (Millipore), following the manufacturer’s instructions. Specifically, 30 embryos from each treatment group were snap-frozen and lysed by sonication in 200 μl of RAS lysis buffer supplemented with 1 μg/ml aprotinin, 1 μg/ml leupeptin, and 1 mM PMSF. 150 μg of total protein lysate was immunoprecipitated with 5 μl of RAS assay reagent (Raf-1 RBD agarose beads) in a 250-μl reaction. 150 μg of control-treated embryo lysate supplemented with 100 mM GTPγS was used as a technical positive control. Following overnight incubation at 4°C, the beads were collected by centrifugation, washed three times in RAS lysis buffer, boiled in 20 μl of 2× Laemmli buffer supplemented with β-mercaptoethanol, and loaded on a 10% polyacrylamide gel. 25 μg of total protein lysate was used as a loading control. RAS-GTP and total RAS levels were detected by western blotting using the mouse monoclonal pan-RAS antibody (anti-RAS clone RAS10) provided and horseradish peroxidase-conjugated secondary antibody, and the signal was detected using ECL chemiluminescence (Thermo Scientific).

### Statistical Analysis

GraphPad Prism 5 and R (v3.0.2; www.r-project.org) software was used for statistical analysis. Tests included one-way and two-way ANOVA, followed by Tukey’s post-test or Bonferroni’s post-test. Data were plotted as the mean, with error bars representing SEM. For data found to be non-normal and with unequal variance across groups, 95% confidence intervals (CIs) of the group means and the difference between group means were estimated by bootstrapping (CIs were bias-corrected and accelerated and based on 10,000 resamplings). Means were considered significantly different when the bootstrapped 95% CI (of the difference between means) did not include zero.

## Author Contributions

Experiments were conceived, performed, and analyzed by P.R.L., C.A., N.J.G., J.D.A., J.Z., Z.Z., and K.P. Experiments were analyzed by R.R.S., J.D.A., and A.P.L. Experiments were conceived, analyzed, supervised, and funded by D.J.P. and E.E.P. The manuscript was written by E.E.P. with comments from all authors.

## Supplemental Information

Document S1. Figures S1–S3

Movie S1. Open-Arena Positioning of Control-Treated Embryos Before and After Disruption, Related to Figure 1

Movie S2. Open-Arena Positioning of Rolipram-Treated Embryos Before and After Disruption, Related to Figure 1

Document S2. Article plus Supplemental Information

## References

[bib1] Anastasaki C., Estep A.L., Marais R., Rauen K.A., Patton E.E. (2009). Kinase-activating and kinase-impaired cardio-facio-cutaneous syndrome alleles have activity during zebrafish development and are sensitive to small molecule inhibitors. Hum. Mol. Genet..

[bib2] Anastasaki C., Rauen K.A., Patton E.E. (2012). Continual low-level MEK inhibition ameliorates cardio-facio-cutaneous phenotypes in zebrafish. Dis. Model Mech..

[bib3] Axelrad M.E., Schwartz D.D., Katzenstein J.M., Hopkins E., Gripp K.W. (2011). Neurocognitive, adaptive, and behavioral functioning of individuals with Costello syndrome: a review. Am. J. Med. Genet. C Semin. Med. Genet..

[bib4] Baillie G.S., MacKenzie S.J., McPhee I., Houslay M.D. (2000). Sub-family selective actions in the ability of Erk2 MAP kinase to phosphorylate and regulate the activity of PDE4 cyclic AMP-specific phosphodiesterases. Br. J. Pharmacol..

[bib5] Baldwin D.S. (2011). Where is the room for improvement in the drug treatment of depression and anxiety?. Hum. Psychopharmacol..

[bib6] Barcellos L.J.G., Ritter F., Kreutz L.C., Quevedo R.M., da Silva L.B., Bedin A.C., Finco J., Cericato L. (2007). Whole-body cortisol increases after direct and visual contact with a predator in zebrafish, *Danio rerio*. Aquaculture.

[bib7] Best J.D., Berghmans S., Hunt J.J., Clarke S.C., Fleming A., Goldsmith P., Roach A.G. (2008). Non-associative learning in larval zebrafish. Neuropsychopharmacology.

[bib8] Blaser R.E., Chadwick L., McGinnis G.C. (2010). Behavioral measures of anxiety in zebrafish (*Danio rerio*). Behav. Brain Res..

[bib9] Burgin A.B., Magnusson O.T., Singh J., Witte P., Staker B.L., Bjornsson J.M., Thorsteinsdottir M., Hrafnsdottir S., Hagen T., Kiselyov A.S. (2010). Design of phosphodiesterase 4D (PDE4D) allosteric modulators for enhancing cognition with improved safety. Nat. Biotechnol..

[bib10] Cachat J., Stewart A., Grossman L., Gaikwad S., Kadri F., Chung K.M., Wu N., Wong K., Roy S., Suciu C. (2010). Measuring behavioral and endocrine responses to novelty stress in adult zebrafish. Nat. Protoc..

[bib11] Cheng Y.F., Wang C., Lin H.B., Li Y.F., Huang Y., Xu J.P., Zhang H.T. (2010). Inhibition of phosphodiesterase-4 reverses memory deficits produced by Abeta25-35 or Abeta1-40 peptide in rats. Psychopharmacology (Berl).

[bib12] Cherry J.A., Davis R.L. (1999). Cyclic AMP phosphodiesterases are localized in regions of the mouse brain associated with reinforcement, movement, and affect. J. Comp. Neurol..

[bib13] Clapcote S.J., Lipina T.V., Millar J.K., Mackie S., Christie S., Ogawa F., Lerch J.P., Trimble K., Uchiyama M., Sakuraba Y. (2007). Behavioral phenotypes of Disc1 missense mutations in mice. Neuron.

[bib14] DeMarch Z., Giampà C., Patassini S., Bernardi G., Fusco F.R. (2008). Beneficial effects of rolipram in the R6/2 mouse model of Huntington’s disease. Neurobiol. Dis..

[bib15] Egan R.J., Bergner C.L., Hart P.C., Cachat J.M., Canavello P.R., Elegante M.F., Elkhayat S.I., Bartels B.K., Tien A.K., Tien D.H. (2009). Understanding behavioral and physiological phenotypes of stress and anxiety in zebrafish. Behav. Brain Res..

[bib16] Engels P., Abdel'Al S., Hulley P., Lubbert H. (1995). Brain distribution of four rat homologues of the Drosophila dunce cAMP phosphodiesterase. J. Neurosci. Res..

[bib17] Griebel G., Holmes A. (2013). 50 years of hurdles and hope in anxiolytic drug discovery. Nat. Rev. Drug Discov..

[bib18] Hansen G., Jin S., Umetsu D.T., Conti M. (2000). Absence of muscarinic cholinergic airway responses in mice deficient in the cyclic nucleotide phosphodiesterase PDE4D. Proc. Natl. Acad. Sci. USA.

[bib19] Hansen R.T., Conti M., Zhang H.T. (2014). Mice deficient in phosphodiesterase-4A display anxiogenic-like behavior. Psychopharmacology (Berl).

[bib20] Heaslip R.J., Evans D.Y. (1995). Emetic, central nervous system, and pulmonary activities of rolipram in the dog. Eur. J. Pharmacol..

[bib21] Himes B.E., Hunninghake G.M., Baurley J.W., Rafaels N.M., Sleiman P., Strachan D.P., Wilk J.B., Willis-Owen S.A., Klanderman B., Lasky-Su J. (2009). Genome-wide association analysis identifies PDE4D as an asthma-susceptibility gene. Am. J. Hum. Genet..

[bib22] Hoffmann R., Baillie G.S., MacKenzie S.J., Yarwood S.J., Houslay M.D. (1999). The MAP kinase ERK2 inhibits the cyclic AMP-specific phosphodiesterase HSPDE4D3 by phosphorylating it at Ser579. EMBO J..

[bib23] Hu W., Lu T., Chen A., Huang Y., Hansen R., Chandler L.J., Zhang H.T. (2011). Inhibition of phosphodiesterase-4 decreases ethanol intake in mice. Psychopharmacology (Berl).

[bib24] Li Y.F., Huang Y., Amsdell S.L., Xiao L., O'Donnell J.M., Zhang H.T. (2009). Antidepressant- and anxiolytic-like effects of the phosphodiesterase-4 inhibitor rolipram on behavior depend on cyclic AMP response element binding protein-mediated neurogenesis in the hippocampus. Neuropsychopharmacology.

[bib25] Li Y.F., Cheng Y.F., Huang Y., Conti M., Wilson S.P., O'Donnell J.M., Zhang H.T. (2011). Phosphodiesterase-4D knock-out and RNA interference-mediated knock-down enhance memory and increase hippocampal neurogenesis via increased cAMP signalling. J. Neurosci..

[bib26] Maurice D.H., Ke H., Ahmad F., Wang Y., Chung J., Manganiello V.C. (2014). Advances in targeting cyclic nucleotide phosphodiesterases. Nat. Rev. Drug Discov..

[bib27] Maximino C., Lima M.G., Olivera K.R., Picanco-Diniz D.L., Herculano A.M. (2011). Adenosine A1, but not A2, receptor blockade increases anxiety and arousal in zebrafish. Basic Clin. Pharmacol. Toxicol..

[bib28] Miao Y., He T., Zhu Y., Li W., Wang B., Zhong Y. (2015). Activation of hippocampal CREB by rolipram partially recovers balance between TNF-alpha and IL-10 levels and Improves cognitive deficits in diabetic rats. Cell Mol. Neurobiol..

[bib29] Millar J.K., Pickard B.S., Mackie S., James R., Christie S., Buchanan S.R., Malloy M.P., Chubb J.E., Huston E., Baillie G.S. (2005). DISC1 and PDE4B are interacting genetic factors in schizophrenia that regulate cAMP signalling. Science.

[bib30] Millar J.K., Mackie S., Clapcote S.J., Murdoch H., Pickard B.S., Christie S., Muir W.J., Blackwood D.H., Roder J.C., Houslay M.D. (2007). Disrupted in schizophrenia 1 and phosphodiesterase 4B: towards an understanding of psychiatric illness. J. Physiol..

[bib31] Nilsson-Ardnor S., Wiklund P.G., Lindgren P., Nilsson A.K., Janunger T., Escher S.A., Hallbeck B., Stegmayr B., Asplund K., Holmberg D. (2005). Linkage of ischemic stroke to the PDE4D region on 5q in a Swedish population. Stroke.

[bib32] Norton W., Bally-Cuif L. (2010). Adult zebrafish as a model organism for behavioral genetics. BMC Neurosci..

[bib33] O'Donnell J.M., Zhang H.T. (2004). Antidepressant effects of inhibitors of cAMP phosphodiesterase (PDE4). Trends Pharmacol. Sci..

[bib34] Pagani M.R., Oishi K., Gelb B.D., Zhong Y. (2009). The phosphatase SHP2 regulates the spacing effect for long-term memory induction. Cell.

[bib35] Perez-Torres S., Miro X., Palacios J.M., Cortes R., Puigdomenech P., Mengod G. (2000). Phosphodiesterase type 4 isozymes expression in human brain examined by in situ hybridization histochemistry and [^3^H]rolipram binding autoradiography. Comparison with monkey and rat brain. J. Chem. Neuroanat..

[bib36] Rauen K.A. (2013). The RASopathies. Annu. Rev. Genomics Hum. Genet..

[bib37] Reneland R.H., Mah S., Kammerer S., Hoyal C.R., Marnellos G., Wilson S.G., Sambrook P.N., Spector T.D., Nelson M.R., Braun A. (2005). Association between a variation in the phosphodiesterase 4D gene and bone mineral density. BMC Med. Genet..

[bib38] Richendrfer H., Pelkowski S.D., Colwill R.M., Creton R. (2012). On the edge: pharmacological evidence for anxiety-related behavior in zebrafish larvae. Behav. Brain Res..

[bib39] Rihel J., Schier A.F. (2012). Behavioral screening for neuroactive drugs in zebrafish. Dev. Neurobiol..

[bib40] Runtuwene V., van Eekelen M., Overvoorde J., Rehmann H., Yntema H.G., Nillesen W.M., van Haeringen A., van der Burgt I., Burgering B., den Hertog J. (2011). Noonan syndrome gain-of-function mutations in NRAS cause zebrafish gastrulation defects. Dis. Model Mech..

[bib41] Rutten K., Basile J.L., Prickaerts J., Blokland A., Vivian J.A. (2008). Selective PDE inhibitors rolipram and sildenafil improve object retrieval performance in adult cynomolgus macaques. Psychopharmacology (Berl).

[bib42] Samatar A.A., Poulikakos P.I. (2014). Targeting RAS-ERK signalling in cancer: promises and challenges. Nat. Rev. Drug Discov..

[bib43] Schnorr S.J., Steenbergen P.J., Richardson M.K., Champagne D.L. (2012). Measuring thigmotaxis in larval zebrafish. Behav. Brain Res..

[bib44] Sheppard C.L., Lee L.C., Hill E.V., Henderson D.J., Anthony D.F., Houslay D.M., Yalla K.C., Cairns L.S., Dunlop A.J., Baillie G.S. (2014). Mitotic activation of the DISC1-inducible cyclic AMP phosphodiesterase-4D9 (PDE4D9), through multi-site phosphorylation, influences cell cycle progression. Cell Signal..

[bib45] Silvestre J.S., Fernandez A.G., Palacios J.M. (1999). Effects of rolipram on the elevated plus-maze test in rats: a preliminary study. J. Psychopharmacol..

[bib46] Siuciak J.A., Chapin D.S., McCarthy S.A., Martin A.N. (2007). Antipsychotic profile of rolipram: efficacy in rats and reduced sensitivity in mice deficient in the phosphodiesterase-4B (PDE4B) enzyme. Psychopharmacology (Berl).

[bib47] Siuciak J.A., McCarthy S.A., Chapin D.S., Martin A.N. (2008). Behavioral and neurochemical characterization of mice deficient in the phosphodiesterase-4B (PDE4B) enzyme. Psychopharmacology (Berl).

[bib48] Staton J.M., Sayer M.S., Hankey G.J., Attia J., Thakkinstian A., Yi Q., Cole V.J., Baker R., Eikelboom J.W. (2006). Association between phosphodiesterase 4D gene and ischaemic stroke. J. Neurol. Neurosurg. Psychiatry.

[bib49] Stewart A., Wu N., Cachat J., Hart P., Gaikwad S., Wong K., Utterback E., Gilder T., Kyzar E., Newman A. (2011). Pharmacological modulation of anxiety-like phenotypes in adult zebrafish behavioral models. Prog. Neuropsychopharmacol. Biol. Psychiatry.

[bib50] Tidyman W.E., Rauen K.A. (2009). The RASopathies: developmental syndromes of Ras/MAPK pathway dysregulation. Curr. Opin. Genet. Dev..

[bib51] Titus D.J., Sakurai A., Kang Y., Furones C., Jergova S., Santos R., Sick T.J., Atkins C.M. (2013). Phosphodiesterase inhibition rescues chronic cognitive deficits induced by traumatic brain injury. J. Neurosci..

[bib52] Viosca J., Schuhmacher A.J., Guerra C., Barco A. (2009). Germline expression of H-Ras(G12V) causes neurological deficits associated to Costello syndrome. Genes Brain Behav..

[bib53] Wolman M., Granato M. (2012). Behavioral genetics in larval zebrafish: learning from the young. Dev. Neurobiol..

[bib54] Wolman M.A., de Groh E.D., McBride S.M., Jongens T.A., Granato M., Epstein J.A. (2014). Modulation of cAMP and Ras signalling pathways improves distinct behavioral deficits in a zebrafish model of neurofibromatosis type 1. Cell Rep..

[bib55] Wu X., Simpson J., Hong J.H., Kim K.H., Thavarajah N.K., Backx P.H., Neel B.G., Araki T. (2011). MEK-ERK pathway modulation ameliorates disease phenotypes in a mouse model of Noonan syndrome associated with the Raf1(L613V) mutation. J. Clin. Invest..

[bib56] Xu Y., Zhang H.T., O'Donnell J.M. (2011). Phosphodiesterases in the central nervous system: implications in mood and cognitive disorders. Handb Exp. Pharmacol..

[bib57] Zhang H.T. (2009). Cyclic AMP-specific phosphodiesterase-4 as a target for the development of antidepressant drugs. Curr. Pharm. Des..

[bib58] Zhang H.T., Huang Y., Jin S.L., Frith S.A., Suvarna N., Conti M., O'Donnell J.M. (2002). Antidepressant-like profile and reduced sensitivity to rolipram in mice deficient in the PDE4D phosphodiesterase enzyme. Neuropsychopharmacology.

[bib59] Zhang H.T., Zhao Y., Huang Y., Dorairaj N.R., Chandler L.J., O'Donnell J.M. (2004). Inhibition of the phosphodiesterase 4 (PDE4) enzyme reverses memory deficits produced by infusion of the MEK inhibitor U0126 into the CA1 subregion of the rat hippocampus. Neuropsychopharmacology.

[bib60] Zhang H.T., Huang Y., Masood A., Stolinski L.R., Li Y., Zhang L., Dlaboga D., Jin S.L., Conti M., O'Donnell J.M. (2008). Anxiogenic-like behavioral phenotype of mice deficient in phosphodiesterase 4B (PDE4B). Neuropsychopharmacology.

[bib61] Zhang X., Li X., Li M., Ren J., Yun K., An Y., Lin L., Zhang H. (2015). Venlafaxine increases cell proliferation and regulates DISC1, PDE4B and NMDA receptor 2B expression in the hippocampus in chronic mild stress mice. Eur. J. Pharmacol..

