# Supplementary material for: MEK Inhibitors Reverse cAMP-Mediated Anxiety in Zebrafish
Source: Chem Biol. 2015 Oct 22;22(10):1335–46. doi: 10.1016/j.chembiol.2015.08.010 (PMC4623357; doi:10.1016/j.chembiol.2015.08.010)
Supplement: Document S1. Figures S1–S3 [file mmc1.pdf]

Chemistry & Biology, Volume *22*

Supplemental Information

MEK Inhibitors Reverse cAMP-Mediated Anxiety in Zebrafish

Pia R. Lundegaard, Corina Anastasaki, Nicola J. Grant, Rowland R. Sillito, Judith Zich, Zhiqiang Zeng, Karthika Paranthaman, Anders Peter Larsen, J. Douglas Armstrong, David J. Porteous, and E. Elizabeth Patton

Supplementary Movies

Movie 1, Related to Figure 1. Open-arena positioning of control treated embryos before and after disruption. The position of DMSO treated zebrafish embryos in a 10-cm Petri dish is disrupted by swirling the water, and recorded for approximately 30 seconds.

Movie 2, Related to Figure 1. Open-arena positioning of rolipram treated embryos before and after disruption. The position of 15μM rolipram treated zebrafish embryos in a 10-cm Petri dish is disrupted by swirling the water, and recorded for approximately 30 seconds.

Supplementary Figures

Figure S1, Related to Figure 1 | Rolipram treatment causes thigmotaxis in zebrafish larvae, before and after distruption.

(A) Thigmotaxis in an open arena is rapidly re-established after disruption. Still images of the video recorded with DMSO and rolipram treated embryos. Zebrafish embryos were treated with 15μM rolipram for one hour, the dish swirled to disrupt the embryo positions, and then followed for 30 seconds by video (See also **Movies 1, 2**). Larvae exhibiting thigmotaxsis are highlighted with orange dots; non-thigmotaxic with blue dots.

Figure S2, Related to Figure 5 | MEKi alone are not anxiolytic in the novel tank test.

Analysis of single adult zebrafish movements and tank area distribution in the novel tank assay. Fish were pre-treated in 200 ml of drug for 20 minutes, transferred to a 3-liter novel tank and recorded for 5 min (Egan et al., 2009). Drug treatment conditions: MEKi: 2μM; buspirone: 10 μM, rolipram: 15μM. **$p<0.01$; n.s. not significant; unpaired two-tailed t-test. Experimental repetitions n=2 with 5 adult fish/per treatment condition. Error bars denote SEM.

Figure S3, Related to Figure 5 | Behavior analysis of *pde4d* mutant zebrafish

(A) Schematic comparison between zebrafish Pde4d compared to PDE4D in humans, mice and *Drosophila*. The asterisks denote the point mutation in the *pde4d-/-* fish from the Sanger Institute, UK.

(B-D) Image and quantification of tracking from the top of the tank. Distance between individual fish in shoal (shoal cohesion) and total distance moved were measured for 5 minutes following 20 minutes of drug treatment. Mutant *pde4d-/-* fish were compared to *pde4d+/+* wild type siblings. Unpaired two-tailed T-test.

(E) Group behaviour test with adult *pde4d-/-* and sibling *pde4d+/+* fish, treated with DMSO or rolipram (15µM). Mutant *pde4d-/-* fish show increased explorative behaviour compared with their wild type siblings that is decreased upon rolipram treatment. Two way ANOVA with Bonferroni post-test, *** $p<0.001$. (Groups of 4 fish, experimental repetitions n=3).

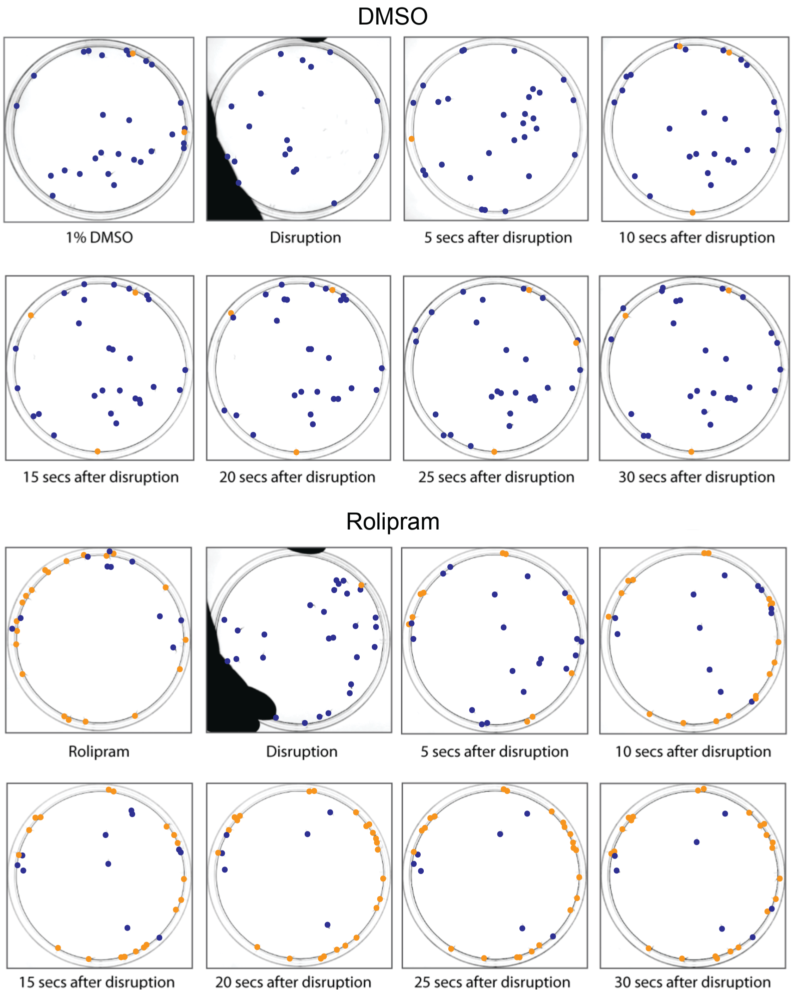

Figure S1 | Rolipram treatment causes thigmotaxsis in zebrafish larvae, before and after disruption.

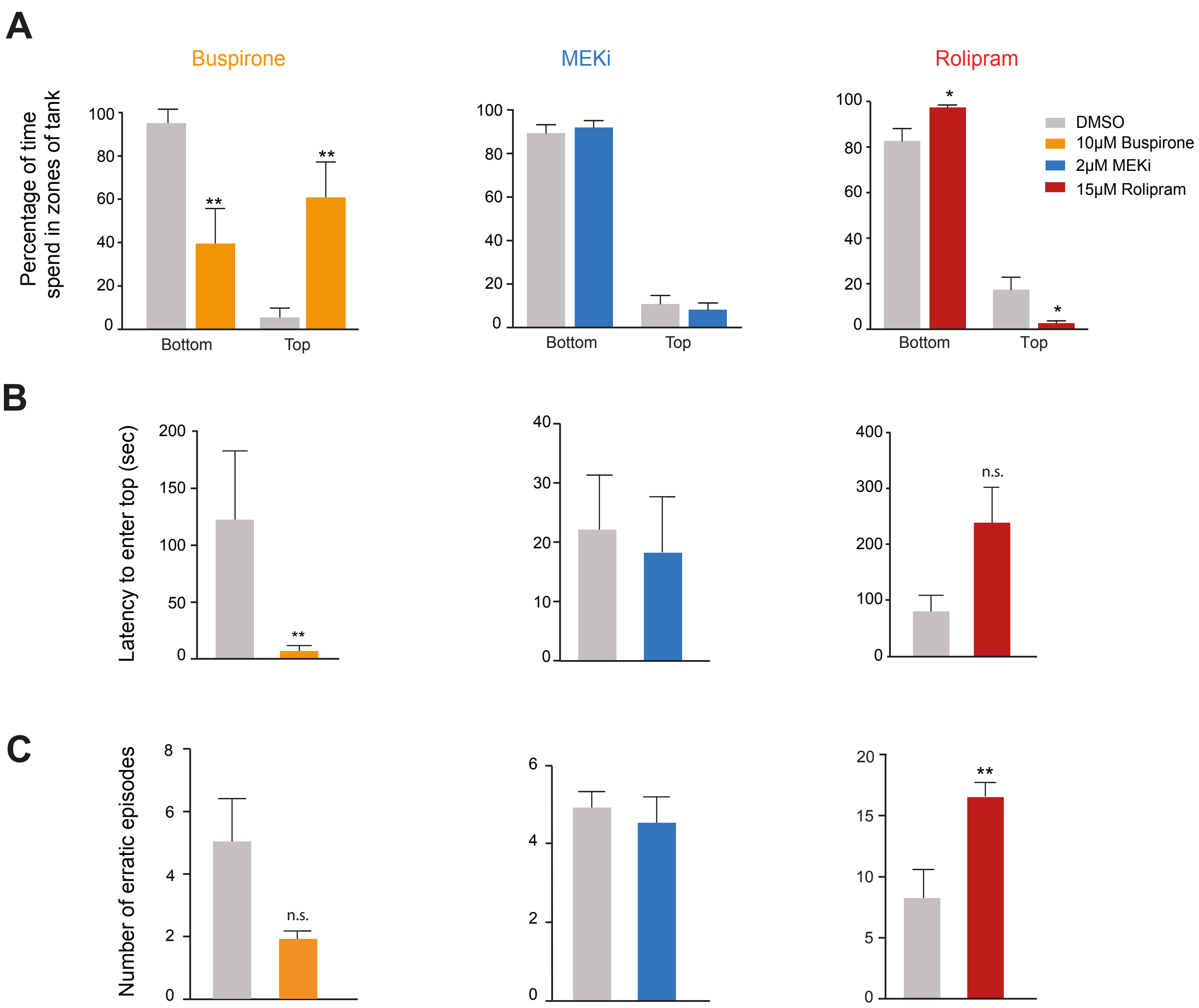

Figure S2 | Novel tank assay analysis for drug treatments

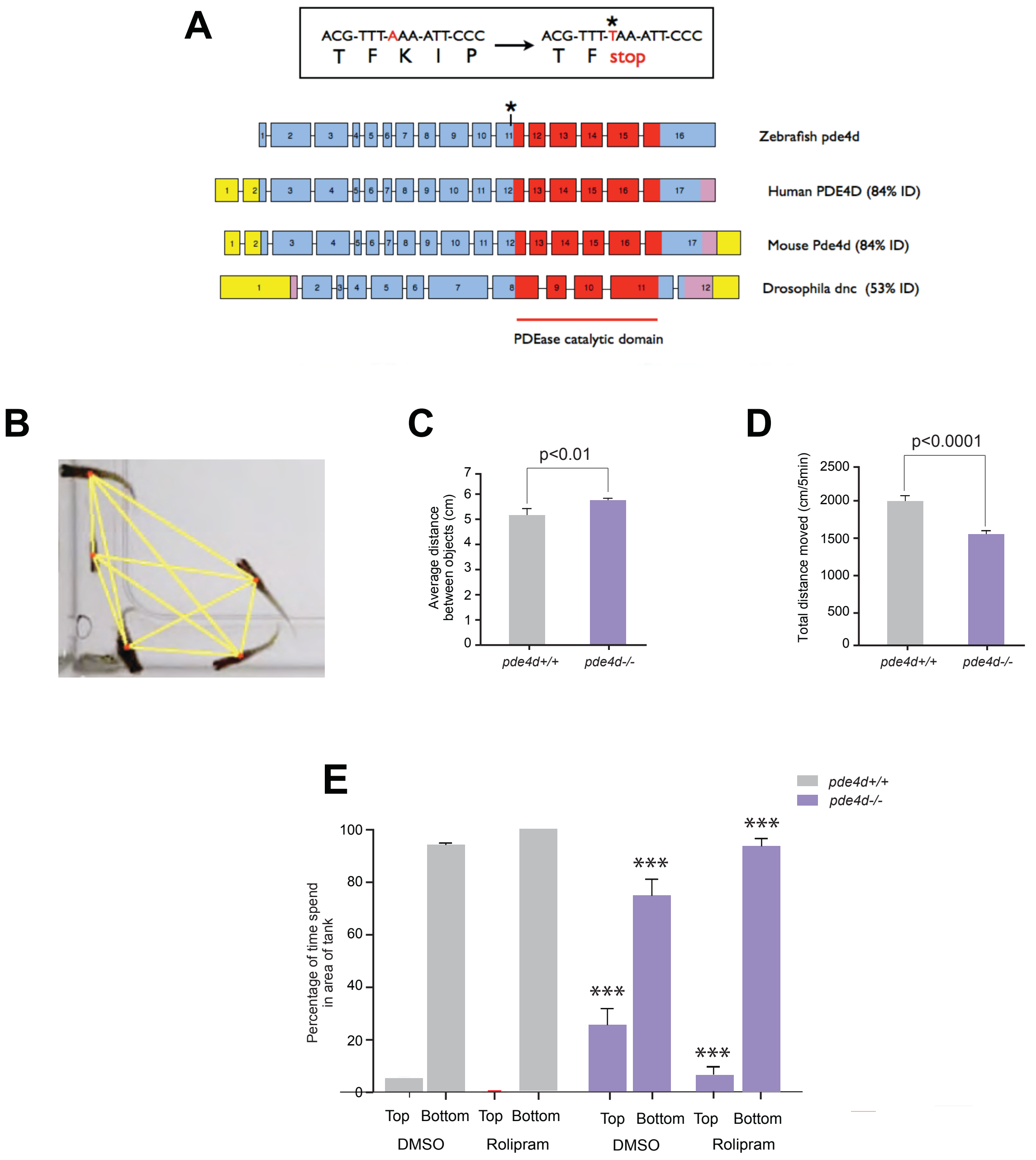

Figure S3 | Behaviour analysis of *pde4d* mutant zebrafish
