# Supplementary figures and images for: MEK Inhibitors Reverse cAMP-Mediated Anxiety in Zebrafish
Source: Chem Biol. 2015 Oct 22;22(10):1335–46. doi: 10.1016/j.chembiol.2015.08.010 (PMC4623357; doi:10.1016/j.chembiol.2015.08.010)

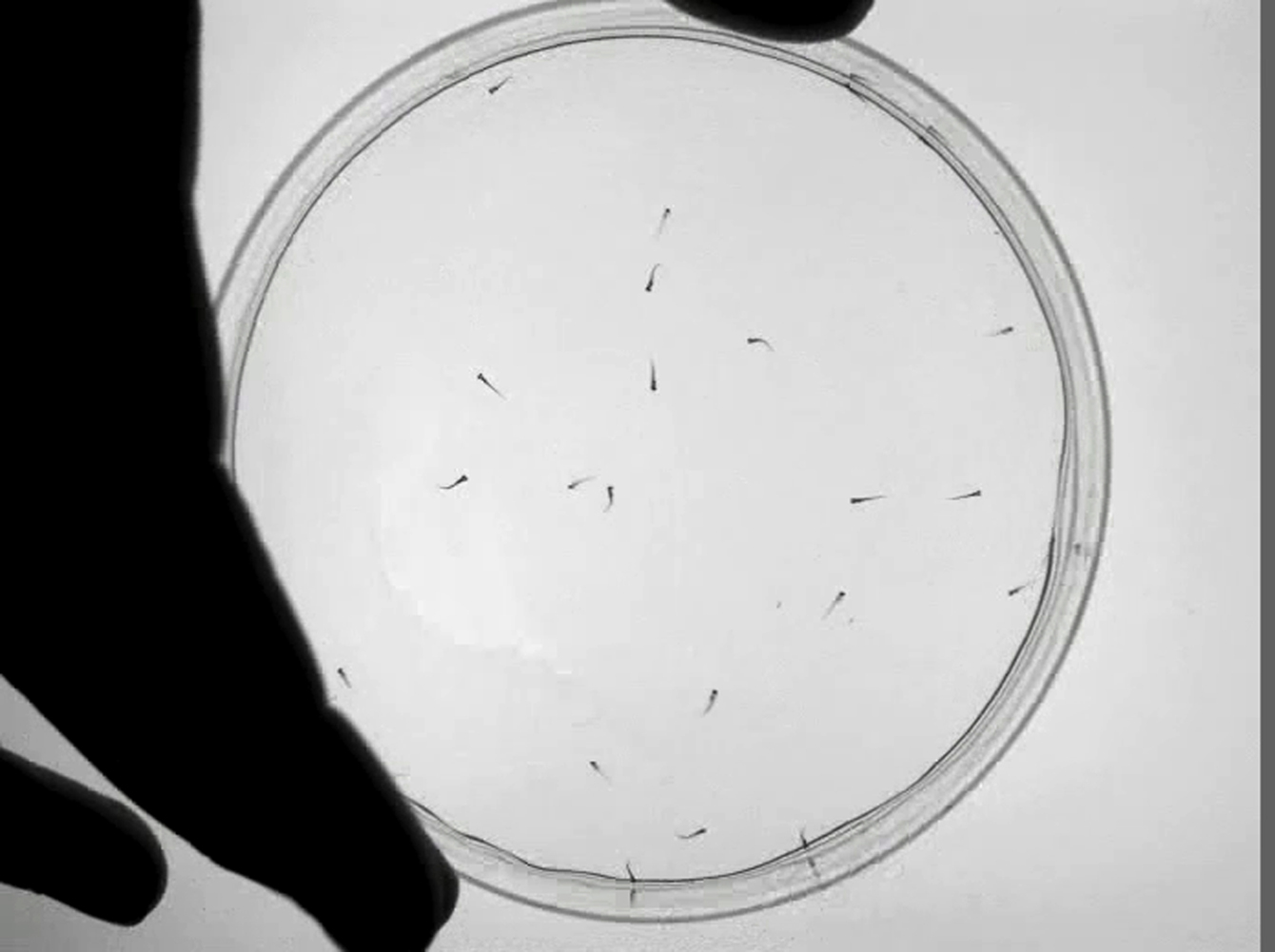

Supplement: Movie S1. Open-Arena Positioning of Control-Treated Embryos Before and After Disruption, Related to Figure 1 [file mmc2.jpg]

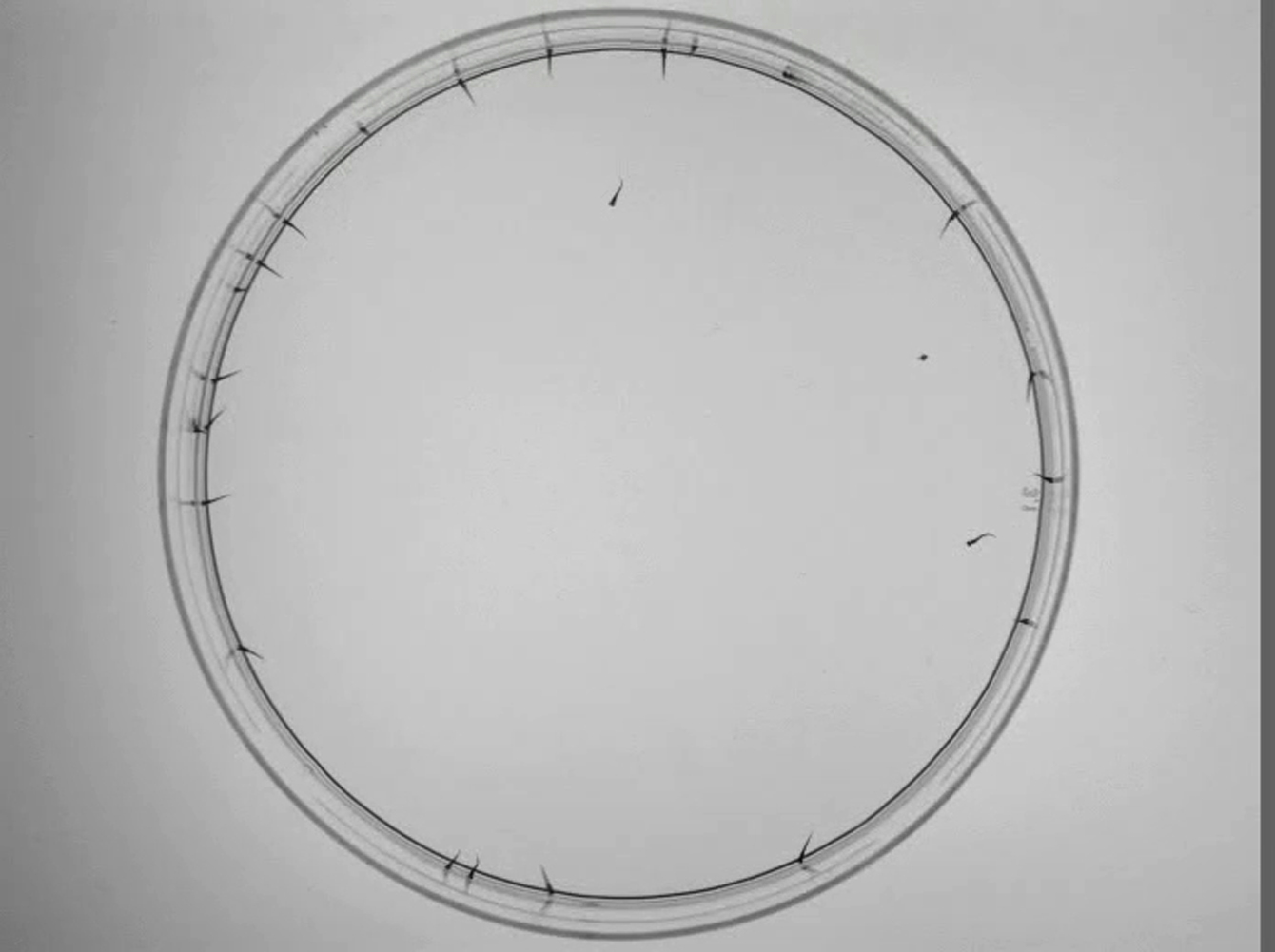

Supplement: Movie S2. Open-Arena Positioning of Rolipram-Treated Embryos Before and After Disruption, Related to Figure 1 [file mmc3.jpg]
